# Multiple omics levels of chronic lymphocytic leukemia

**DOI:** 10.1038/s41420-024-02068-2

**Published:** 2024-06-21

**Authors:** Aleksander Turk, Eva Čeh, George A. Calin, Tanja Kunej

**Affiliations:** 1 Clinical Institute of Genomic Medicine, University Clinical Centre Ljubljana, Ljubljana, Slovenia; 2 Department of Animal Science, Biotechnical Faculty, University of Ljubljana, Ljubljana, Slovenia; 3 Department of Translational Molecular Pathology, Division of Pathology, MD Anderson Cancer Center, University of Texas, Houston, TX 77030 USA

**Keywords:** Cancer genetics, Cancer genomics

## Abstract

Chronic lymphocytic leukemia (CLL) is a lymphoproliferative malignancy characterized by the proliferation of functionally mature but incompetent B cells. It is the most prevalent type of leukemia in Western populations, accounting for approximately 25% of new leukemia cases. While recent advances, such as ibrutinib and venetoclax treatment have improved patient outlook, aggressive forms of CLL such as Richter transformation still pose a significant challenge. This discrepancy may be due to the heterogeneity of factors contributing to CLL development at multiple -omics levels. However, information on the omics of CLL is fragmented, hindering multi-omics-based research into potential treatment options. To address this, we aggregated and presented a selection of important aspects of various omics levels of the disease in this review. The purpose of the present literature analysis is to portray examples of CLL studies from different omics levels, including genomics, epigenomics, transcriptomics, epitranscriptomics, proteomics, epiproteomics, metabolomics, glycomics and lipidomics, as well as those identified by multi-omics approaches. The review includes the list of 102 CLL-associated genes with relevant genomics information. While single-omics studies yield substantial and useful data, they omit a significant level of complex biological interplay present in the disease. As multi-omics studies integrate several different layers of data, they may be better suited for complex diseases such as CLL and have thus far yielded promising results. Future multi-omics studies may assist clinicians in improved treatment choices based on CLL subtypes as well as allow the identification of novel biomarkers and targets for treatments.

## Facts

- Many CLL studies still focus on a single-omics level.
- CLL is a highly complex disease and may require an integrated, multi-omics approach.
- Factors contributing to CLL span multiple omics levels, including genomic, epigenomic, transcriptomic, epitranscriptomic, proteomic, epiproteomic, metabolomic, glycomic, lipidomic and multi-omic levels.

## Open questions

- Can a multi-omics perspective enhance our understanding of CLL pathomechanism and aid in developing more effective treatment options?
- How might multi-omics assist clinicians in guiding therapeutics for refractory or relapsed CLL cases?
- Will multi-omics facilitate the identification of novel therapeutics targets for CLL treatment, either through repurposing or developing new therapies?
- What advances can be expected in understanding the molecular mechanisms underlaying Richter’s Transformation through multi-omics studies?
- Could combined therapeutics prove beneficial in treating relapsed CLL cases?
- Are CLL biomarkers expected to involve multiple omics components?

## Introduction

Chronic lymphocytic leukemia (CLL) is a malignancy characterized by the proliferation of mature-appearing but functionally incompetent B cells [1]. As the disease often progresses slowly, this uncontrolled cell proliferation is often asymptomatic at the time of diagnosis [1, 2]. When symptoms appear, they usually include weight loss, fatigue, lymphadenopathy, anemia, thrombocytopenia, hepatomegaly and splenomegaly [1]. However, due to the slow progression of the disease, patients with low-risk CLL and asymptomatic patients do not require treatment, as early intervention does not yield survival benefits [3]. Instead, a watch-and-wait strategy is used for the CLL’s early stages, which shifts if the disease progresses [4]. Approximately 50% of patients need to start treatment within 5 years of the initial diagnosis [5]. A more expedient need for treatment arises if CLL evolves into a very aggressive form, such as Richter transformation (RT) (also known as Richter syndrome) [6].

CLL is the most prevalent type of leukemia in Western countries, accounting for approximately 25% of all new leukemia cases [7]. In a study that included data from 204 countries, over 100,000 people have been diagnosed with CLL in 2019, a marked increase since 1990 [8]. The disease primarily affects older adults, with the median age at the time of diagnosis between 65 and 70 years [9]. CLL is not strictly limited to this demographic however, in Europe and the USA 5–11% of patients are younger than 50–55 years at the time of diagnosis [10]. Environmental risk factors, such as exposure to certain herbicides or benzene may contribute to disease development, although these results are inconsistent [11, 12]. Despite this, genetic factors appear to play a stronger role in the risk of CLL development compared to environmental effects [13]. Approximately 15–20% of patients with CLL are related to someone with CLL or another lymphoproliferative disorder [14].

Multiple factors from various omics levels have been associated with CLL. Factors on the DNA level include sequence variants in genes (e.g. *NOTCH1*, *TP53* and *FBXW7*) [15], chromosomal aberrations (such as trisomy 12) [16] and copy number variants (*miR15A* and *miR16-1* deletions) [17]. However, associated factors are not limited to the genomic level. The expression of many genes associated with CLL is epigenetically regulated via DNA methylation. For example, *ZAP70, TP63*, *NFATC1* and others have been found to be upregulated as a result of hypomethylated DNA in some CLL cases [18]. Chromatin status influences gene expression by determining the accessibility of DNA for the cell’s transcription apparatus. CLL-specific aberrant changes to chromatin features have been observed, such as changes around enhancer and promoter elements [19]. Disease-associated factors have also been identified on other omics levels, such as proteomics [20], transcriptomics [21] and metabolomics [22].

As more research is conducted on CLL, it is becoming increasingly apparent that there is no singular cause for disease development. Some factors, such as immunoglobulin heavy chain (IGHV) mutation status, are known biomarkers and have prognostic value [23]. Despite this, current prognostic model scores cannot predict the evolution of CLL in patients with absolute precision [24]. As a highly complex disease with a plethora of contributing factors, single-omics approaches may not be best suited for understanding the etiology of CLL. Instead, a multi-omics approach may yield better insights into its pathomechanism and could be instrumental in the development of improved prognostic or therapeutic methods. However, the fragmented nature of CLL information from different omics levels makes this challenging. In order to address this, we aggregated information on CLL from multiple omics levels and presented in the present review.

**Fig. 1:**
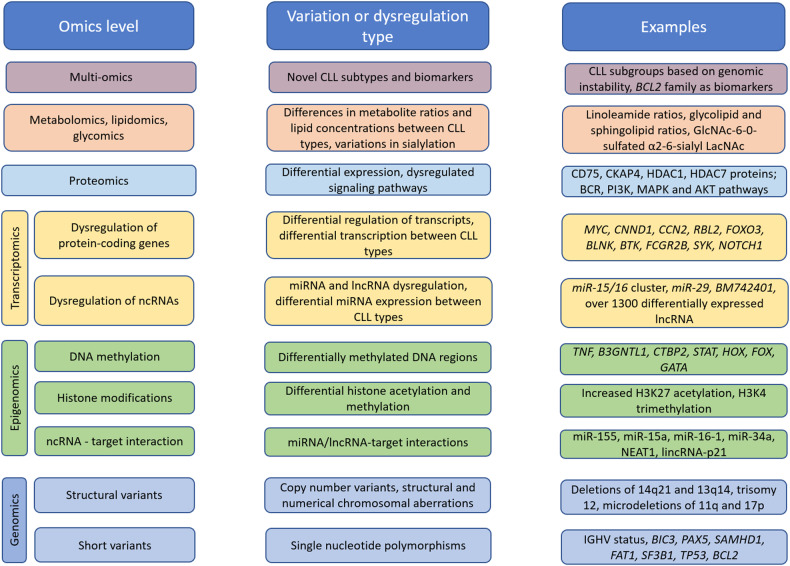
Graphical abstract of the review. The figure is split into the omics level reviewed in the present study, the type or variation or dysregulation found in literature on the respective level and examples of affected factors.

In this review, we obtained reported data on factors associated with CLL on different omics levels, including genomics, epigenomics, transcriptomics, proteomics, metabolomics, glycomics, lipidomics and multi-omics. Additionally, due to the differences between CLL and RT, an overview of the reported data on the multiple omics levels of RT is included as a separate section. A graphical abstract of the review is presented in Fig. 1.

## Genomics

Factors associated with CLL have been extensively studied on the DNA level, which falls under the purview of genomics. Various DNA alterations have been associated with CLL, including single nucleotide polymorphisms (SNPs), microdeletions, copy number variants (CNVs), chromosome aberrations, and others. In the literature and databases, sequence variants are frequently divided into short and structural variants using a threshold of around 50 base pairs.

### Short variants

Short genetic variants include variations in the DNA sequence that are under 50 base pairs in length – these commonly include single nucleotide variants (SNVs) and short insertions or deletions. Sequence variants in CLL can be valuable prognostic markers for patients, as the mutation status of CLL genes can be indicative of disease progression. The IGHV region encodes the V, D, J and C segments required to form the immunoglobulin heavy chain [25]. A mutated IGHV status is indicative of better prognosis, though the reason for this is unknown. It has been proposed that this may be caused by the utilization of either a high-fidelity or low-fidelity DNA repair mechanism, which depends on the speed of cell proliferation – rapidly dividing cells use a high-fidelity homology-directed DNA repair apparatus, while slowly dividing cells use an inefficient, low-fidelity end-joining repair mechanism. As such, a low IGHV mutation rate can be observed in rapidly dividing cells whereas slowly dividing cells will typically have a higher IGHV mutation rate [26]. Regardless of the cause, mutated (M-CLL) vs unmutated IGHV CLL (U-CLL) subtypes display distinct differences in the mutation status of other CLL genes. U-CLL cases more commonly have mutations in *IKZF3* [27], which is a transcription factor needed for regulating B cell differentiation and proliferation [28]. Additionally, in U-CLL cases, mutations were detected in the CLL-associated genes *BIRC3*, *PAX5* and *SAMHD1* [27].

Mutations in genes such as *ATM, NFKBIE, NOTCH1, SF3B1* and *TP53* are among the most well known to be associated with CLL. These changes can convey significant variations to a patient’s prognosis. Some mutations, such as in *RPS15*, are enriched in aggressive CLL cases [29]. However, mutations in certain genes are also associated with chemorefractory CLL (CR-CLL), which does not respond to chemotherapy. *FAT1*, as well as *SF3B1* and *TP53*, have been associated with fludarabine refractoriness [30–32]. Similarly, acquired mutations in the BH3-binding domain of *BCL2* have been associated with resistance to the BCL2 antagonist venetoclax [33]. SNPs have also been associated with CLL - a series of genome-wide association studies (GWAS) have so far identified over 40 risk SNPs in various CLL risk loci [34–39]. Interestingly, the majority of these loci were mapped to regulatory regions [40].

### Structural variants

Structural variants (SVs) are generally defined as a region of DNA over 50 bp which includes insertions, deletions (commonly called copy number variants; CNVs) or inversions, though older definitions required a region of approximately 1 kb or larger to be considered an SV [41]. Several SVs have been associated with CLL. In a study of two U-CLL patients, Fillerova et al. [42] detected large deletions of 17.5 kbp in 9q21 and 7.1 kbp in 14q21 in both patients. Additionally, the patients also had intra-chromosomal translocations in the 13q14 region, among other large somatic SVs [42]. 13q14 deletions are common in CLL and are found in approximately 50% of all cases [43, 44]. This deletion affects the prognosis and can be divided into two types [43]. Type one constitutes the deletion of *miR15A/16-1*, which results in increased multiplication of B lymphocytes [43]. In type two, the tumor suppressor gene *RB1* is deleted [43], which can lead to the development of other types of cancers [45]. Despite this, other SVs are also associated with CLL, as shown by Burns et al. [27]. In 30 of the 46 patients included in the study, 79 interchromosomal translocations were detected [27]. Additionally, their results further support that sites of kataegis (localized hypermutations) colocalize with structural rearrangements [27].

Trisomy 12 (+12) is present in CLL in about 20% of cases, but its pathophysiological role in the disease is not well known [46]. Patients with +12 CLL are significantly more likely to have unmutated IGHV status compared to subgroups with 13q deletions or those with a normal karyotype [47]. In a cohort of 39 +12 CLL cases, about 39% of patients had microdeletions of *miR15A/16-1* [48]. These microdeletions also occurred in patients with 11q and 17p deletions. It has thus been suggested that loss of *miR15A/16-1* at 13q cooperates with other chromosomal alterations in CLL [48]. Additionally, +12 CLL cases also have other unique morphological, immunophenotypic and genetic characteristics [46]. *TP53* is rarely mutated [46], however *NOTCH1* mutations are frequent, appearing in about 34% of +12 CLL patients [49].

## Epigenomics

Epigenomics is the study of reversible modifications including four main mechanisms: DNA methylation, histone modifications, chromatin remodeling and non-coding RNA (ncRNA)-mediated regulation.

### DNA methylation

DNA methylation regulates gene expression by preventing transcription factors from binding to promoters. While many studies have been conducted on the DNA level, the field of epigenomics has made significant progress in CLL. Whole-genome DNA methylation analyses have identified three distinct CLL subgroups: naïve B cell-like CLL (n-CLL), intermediate CLL (i-CLL) and memory B cell-like CLL (m-CLL). These three subgroups showed differential levels of IGHV mutation and different clinical features as well as time to first treatment and overall survival. The model is based on the methylation status of five biomarkers – the *TNF, B3GNTL1* and *CTBP2* genes, the *SCARF1* promoter region and an intergenic region on chromosome 14 [50]. The results of another study also showed that M-CLL and U-CLL cases are epigenetically different, with the two types being distinguishable by 3265 differentially methylated CpG sites. The epigenetic signatures of U-CLL resembled naïve B cells (NBC) and CD5^+^ NBCs, whereas M-CLL cases more closely resembled memory B cells (MBC) [51]. The clinical significance of these CLL epigenetic subgroups has also been validated in clinical data, as m-CLL shows a favorable response to fludarabine-cyclophosphamide-rituximab (FCR) regimen [52].

Epigenetic alterations, while associated with CLL, emerge before the actual disease onset and persist throughout disease stages [53]. At a cohort level, DNA methylation levels in CLL cells appear to generally undergo limited changes [53], though certain patients show significant epigenetic evolution, specifically after relapse [54]. CLL cells are characterized by extensive hypomethylation compared to MBC, though some hypermethylation occurs [51, 54]. These hypomethylation events cluster mainly in gene bodies and heterochromatin regions, while hypermethylation mainly occurs in promoters and Polycomb-related regions [54]. Certain genomic regions relevant to CLL biology become hypomethylated prior to treatment as well as after a relapse. These regions are enriched for the binding sites of certain transcription factor families, including *GATA, STAT, HOX* and *FOX* [54].

### Histone modifications

Histones play a role in gene expression by packing DNA into the transcriptionally inactive (heterochromatin) or active (euchromatin) state. This is regulated by reversible histone modifications, such as methylation or acetylation of histone tails, which affect the transcription factors’ access to DNA [55]. Changes to histone modification patterns have been associated with multiple malignancies, including CLL. An analysis of H3K27 acetylation (H3K27ac) revealed that 297 super-enhancers were differentially regulated in CLL compared with normal B cells [56]. This acetylation was increased near genes necessary for lymphocyte proliferation and differentiation, such as *BCL2, LEF1* and *CTLA4* [57–59]. Beside H3K27ac, H3K27 trimethylation (H3K27me3) and H3K4 trimethylation (H3K4me3) were also associated with CLL. High H3K27me3, and low H3K4me3 and H3K27ac, were associated with uniform gene silencing in normal B cells, but were associated with variable expression in CLL [56].

Many proteins are required for the transfer of methyl or acetyl groups onto histone tails, and some have been associated with CLL. EZH2 is a subunit of the Polycomb repressive complex 2 (PRC2), a complex with histone methyltransferase activity, mainly responsible for H3K27me3 [60]. Overexpression of *EZH2* was found in U-CLL patients, which was associated with high H3K27me3 levels. *EZH2* overexpression was also associated with increased CLL cell viability, while lower expression resulted in apoptosis. Treatment with EZH2 inhibitors led to decreased H3K27me3 levels and induced apoptosis, making it a potential therapeutic target for certain aggressive CLL types [61]. Mutations in other genes responsible for chromatin remodeling have also been associated with CLL. These include *ARID1A* [62], *CHD2* [63] and *SETD2* [64], which have been reported in approximately 2%, 5% and 4% of CLL cases respectively.

### Non-coding RNA-target interaction

Non-coding RNAs regulate a large proportion of biological processes within a cell, such as cell signaling, development and differentiation. Some hold functions in translation, such as tRNA, but others are a part of epigenetic regulatory mechanisms. The latter RNAs are generally divided into short (sncRNA) or long (lncRNA), depending on whether their transcripts are over or under 200 nucleotides. MicroRNAs (miRNA) are a class of sncRNAs that silence gene expression on a post-transcriptional level [65]. miR-155, which plays a role in regulating gene expression in B cells [66], shows increased expression in CLL, but is barely present in healthy samples [67]. *miR-15a* and *miR-16-1* are located on the commonly deleted region 13q14. They directly negatively regulate *BCL2*, an important oncogene, thus their down-regulation leads to an increase of BCL2 levels [68]. miR-34a has been shown to influence BCL2 expression [69], and this interaction has been shown to be important for regulating apoptosis [70]. miR-34a has also been shown to be frequently downregulated in CLL [71].

**Fig. 2:**
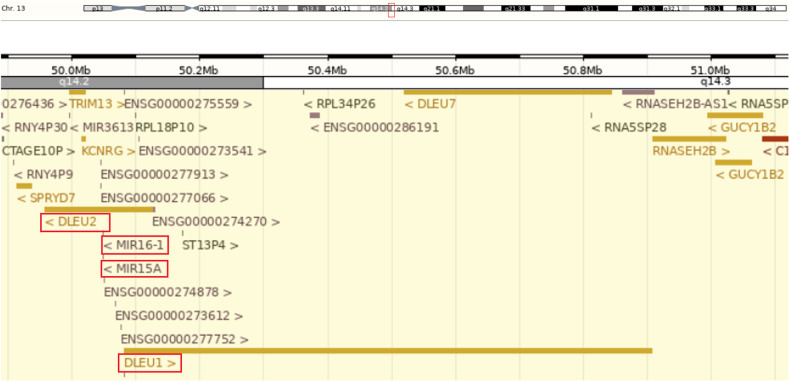
Genomic organization of the chromosome 13q14 with CLL-associated genes: two miRNA genes (*miR15A*, *miR16-1*) and two lncRNAs (*DLEU1* and *DLEU2*). Figure obtained from the Ensembl genome browser.

LncRNAs are involved in transcriptional and post-transcriptional regulation and have been proposed as a diagnostic tool for certain cancers [72]. Some lncRNAs have been shown to function as tumor suppressors [73]. In CLL, a functional P53 has been shown to induce the NEAT1 and lincRNA-p21 lncRNAs, which did not occur with a mutated P53. This is relevant as the induction of NEAT1 and lincRNA-p21 are correlated with apoptosis after DNA damage [74]. Additionally, the common 13q14 deletion also results in the loss of *DLEU1* and *DLEU2* [75], which have been associated with tumor suppression regulation via NF-kB interactions [76]. Figure 2 (sourced from the Ensembl genome browser [77]) illustrates genomic organization of the chromosome 13q14 with CLL-associated genes: two miRNA genes (*miR15A*, *miR16-1*) and two lncRNAs (*DLEU1* and *DLEU2*).

## Transcriptomics

While genomics is concerned with studying diseases at the DNA level, transcriptomics is dedicated to studying the RNA transcripts of genes. This is an important distinction, as many factors can influence whether a DNA sequence is actually transcribed. The complete set of a cell’s transcripts, the transcriptome, also differs between various cell types. This difference also extends to normal and cancerous cells, with multiple transcriptomic distinctions occurring in cancers.

### Dysregulation of protein-coding genes

Though causes may vary, transcriptional dysregulation of protein-coding genes is inevitably seen in cancers, including CLL [78]. This differential transcription alters the functioning of the cell and contributes to the disease phenotype. In CLL, BCR signaling plays an important role in pathogenesis, contributing to the survival and growth of malignant B cells [79]. Pede et al. [80] investigated CLL cell response to BCR stimulation. They found that BCR activation led to increased expression of *MYC*, *CCND1, CCN2, RBL2, FOXO3* and others associated with either cell cycle initiation, progression or survival. They concluded that part of the transcriptional profiles linked to *IGVH* mutation status may not be cell intrinsic, but rather a consequence of stimulation by BCR [80].

In-depth transcriptome analyses provide a view of the levels of transcription occurring within the cell. Ferreira et al. [81] analyzed the transcriptome of 219 CLL patients and found large transcriptional differences between normal lymphocytes and CLL cells. They found that in CLL, 13.6% of the human genome was covered by RNA-seq reads, while in normal cells the average was 10.5% [81]. 1089 genes were differentially transcribed between normal and CLL samples. Among them, genes in the BCR, JAK-STAT signaling and cytosolic DNA sensing pathways were particularly affected. The highly upregulated genes in the BCR signaling pathway included *BLNK, BTK, FCGR2B* and *SYK* [81]. In the BCR pathway, *FCGR2B* encodes the receptor, *SYK* is responsible for signal initiation, while *BLNK* and *BTK* are responsible for signal propagation [81]. On the other hand, six genes from the NF-κB pathway were significantly downregulated. Furthermore, hierarchal clustering revealed two transcriptionally distinct subgroups in the CLL samples, referred to as C1 and C2. These subgroups were independent of *IGHV* mutational status and, together with the mutation status of this region, were the only independent variables in predicting time to treatment [81]. The C1/C2 groups showed markedly different clinical outcomes, with C2 patients having a more aggressive disease course. The C2 group showed shared genetic and pathway up-regulations with CLL cells obtained from the lymph node, which was speculated to be attributable to the lymph node microenvironment influencing the differential gene expression between C1 and C2. Additionally, the C2 group showed significant enrichment in upregulated genes that are upregulated after BCR stimulation by IgM antigens [81].

Other extensive transcriptome analyses have also been carried out. Griffen et al. [82] conducted a multivariate transcriptome analysis on 203 CLL patients, focusing on relapses. Between Binet stage A patients with and without relapse, 1703 genes were downregulated and 1250 were upregulated. Some CLL biomarkers, such as *ATM, CXCR5* and *ZAP70* were significantly differentially expressed between the two groups. However, no differential expression was detected for *BTK, BCL2, CD38, MCL1, NOTCH1, SF3B1* and *TP53*. Additionally, they identified 13 distinct subnetworks (modules) of dysregulated protein-coding genes, 8 of which were correlated with CLL relapse – 5 with positive correlation, 3 with negative correlation. Certain hub genes within these modules have consequently been proposed as potential novel therapeutic targets or clinical markers. These include: *ARHGAP27P2, C1S, CASC2, CLEC3B, CRY1, CXCR5, FUT5, MID1IP1*, and *URAHP* [82]. Sbarrato et al. [83] showed that CLL B cells hold a ribosome-related signature with reduced expression of factors that modify ribosomal rRNA, including *DKC1*. Furthermore, they show that decreased *DKC1* expression is a prognostic factor correlating with poor overall survival following treatment. They hypothesize that low *DKC1* expression could lead to ribosomal protein imbalance, influencing the B cell response to their microenvironment [83].

### Dysregulation of non-coding RNAs

Many transcription dysregulations are known in CLL, but they are not only limited to protein-coding genes - they also commonly affect ncRNAs. Relevant to cancer – the diversity of ncRNAs functions means that they have been identified as both oncogenic drivers and tumor suppressors. Their differential transcription has been observed in the majority of cancer types [84].

A single miRNA can regulate multiple genes [85], thus its transcriptional dysregulation can alter expression levels of several targets, including genes involved in the progression and development of cancer cells [86]. A large number of other miRNAs have also been found to be dysregulated in CLL, including miR-21 [87], miR-155 [88], miR-181b [89], miR-192 [90], miR-338-3p [89] and miR-342-3p [91]. Related miRNAs, miR-34b and miR-34c, are located on the commonly deleted region 11q23 and are also frequently downregulated in CLL [92]. The most common miRNA dysregulation occurs with *miR-15a* and *miR-16-1*. They are downregulated in about 66% of CLL cases [44], and both target *BCL2* [93] and *MCL1* [94]. Increased *miR-15a/miR-16-1* expression levels are inversely correlated with *BCL2* and *MCL1* expression, thus affecting apoptosis [93, 95]. However, when *miR-15a/miR-16-1* are downregulated, *BCL2* is left unregulated, causing reduced apoptosis [93]. Interestingly, isoforms of MCL1 can either inhibit or promote apoptosis [96], meaning that differential expression of the many miRNAs that regulate it [95] can have diverse effects on apoptosis. Other genes affected by the *miR-15a/miR-16-1* cluster include *BAZ2A, RNF41, RASSF5, MKK3* and *LRIG1*, which were upregulated in CLL patients with low *miR-15a/miR-16-1* expression [97]. The miR-16 family targets the transcripts of several genes that play a role in cell cycle regulation, including *E2F7, CDC25A, CHEK1, WEE1* and *CCNE1* [98]. However, there is functional redundancy in miR-16 family members. It has been suggested that this miRNA family functions like gene expression micromanagers rather than a classical tumor suppressor gene [98].

Despite their common occurrence, miRNA dysregulations in CLL are not limited to the *miR-15/16* cluster [99]. In CLL patients with chromosome 17p deletions, *miR-21*, *miR-34a*, *miR-155* and *miR-181b* were differentially expressed alongside *mir-15a*. Additionally, *miR-21* were significantly higher with lower predicted overall survival and a poor prognosis [87]. Beside mere dysregulations in CLL patients, lower *miR-181b* expression levels have been shown to be able to distinguish indolent and aggressive cohorts and have been proposed as a biomarker for disease progression [100]. miRNA dysregulations have also been associated with lower treatment effectiveness. Higher expression levels of *miR-21, miR-148a* and *miR-222* have been associated with fludarabine-resistant CLL [71]. Similarly, lower expression levels of *miR-34a* have been associated with fludarabine refractory CLL [101].

*miR-29* is downregulated in aggressive CLL, which may contribute to pathogenesis through the overexpression of one of its regulatory targets – *TCL1*. Similarly, *miR-181* is also underexpressed in CLL cells and has *TCL1* as its predicted target [102]. In a study of 228 patients with CLL, significantly higher *miR-155* expression was found in those who did not achieve complete response to treatment. Additionally, as B cells progress towards CLL cells, their levels of *miR-155* also increase significantly [103]. *miR-155* regulates the expression of *SHIP1* - a part of the B cell receptor (BCR) signaling pathway, which is responsible for cell survival, proliferation, apoptosis, differentiation and other functions [88, 104]. The *miR-17/92* cluster has also been associated with CLL and other lymphoid malignancies. The results of a study on murine models overexpressing *miR-17/92* in B cells showed that 80% of transgenic mice developed a B cell malignancy with CD19 + B cell expansion [105].

While many miRNAs are associated with CLL, they are not the only class of ncRNA relevant to the disease. Several long non-coding RNAs (lncRNAs) have also been found to be dysregulated in CLL. In comparison to healthy controls, CLL samples displayed hypermethylation and hypomethylation of the *CRNDE* and *AC012065.7* promoters respectively. Expression of *CRNDE* and *AC012065.7* have been positively correlated with the expression of *GDF7* and *IRX5* [106]. *MALAT1* is overexpressed in both CLL [107] and lung cancer [108]. The tumor suppressor lncRNA BM742401 (GATA6-AS1) has been found to be inactivated by DNA methylation in CLL samples and its promoter is fully methylated in CLL cell lines [109]. LncRNAs have many mechanisms of action, one of which is sequestering miRNA – this allows lncRNAs to control the regulatory functions of miRNAs by inhibiting their ability to target mRNA. In this way *HULC* downregulates *miR-372* and *miR-200a-3p*, and is itself upregulated in CLL [110].

Tschumper et al. [111] studied lncRNA expression in the Rai stage 0/I U-CLL patients based on disease progression. In patients who experienced disease progression in under 2 years, versus those who did not experience progression in over 5 years, over 1300 lncRNAs were notably differentially expressed [111]. While a causative relationship has not been established between lncRNAs and CLL, it is clear they should not be overlooked in future research.

Recently, developments have also been made in the field of epitranscriptomics in CLL. Zhang et al. [112] showed that CLL cells display differential N^6^-methyladenosinemethylation peaks in RNA. A total of 1836 significantly changed peaks were detected, with 1519 significantly upregulated and 317 significantly downregulated peaks [112]. Gassner et al. [113] studied RNA editing, namely the conversion from adenosine to inosine in CLL. Results showed substantially altered RNA editing profiles in CLL compared to controls and RNA editing patterns prognostically relevant in CLL [113].

## Proteomics

Like in many other malignancies, proteins play an important role in CLL. The proteome refers to the complete set of proteins produced by an organism, and is studied by the field of proteomics. Advances in analytic approaches within the past few decades have allowed for extensive studies of disease-associated changes within the proteome. These studies enable multiple novel observations, such as comparing expression levels of thousands of proteins in case-control studies, protein localization, protein-protein interactions, their involvement in metabolic pathways and many others.

Johnston et al. [114] performed isobaric labeling and mass spectrometry to analyze 14 CLL B cell samples and compare their protein makeup to healthy controls. Of the 8694 identified proteins, 544 were significantly overexpressed in CLL samples, unrelated to disease subtype. While known CLL hallmarks were identified, the more relevant result is the overexpression of previously unrecognized surface proteins, such as CD75, CKAP4, PIGR, TMCC3, ATP2B4, CLEC17A and LAX1, where the latter three were notable for being involved in BCR signaling [114]. Additionally, other potential targets were also identified, based on existing drug and inhibitor knowledge from Ingenuity Pathway Analysis (IPA). These include HDAC1, HDAC3, HDAC7, HMOX1, HMOX2, MAPK8, MAPK13 and WEE1 [114]. Similarly, Meier-Abt et al. [115] performed mass spectrometry on 117 CLL samples. However, the results showed that major determinants of gene expression were trisomy 12 and IGHV status, with 1055 and 542 differentially expressed proteins respectively. In trisomy 12, the results of a gene enrichment analysis suggest BCR, PI3K, MAPK and AKT signaling as a tumor driver. Additionally, the study included drug response as a parameter, and linked STAT2 protein expression with patients’ response to kinase inhibitors [115].

Various pathways have been implicated in CLL pathogenesis, such as the BCR signaling pathway. While tightly regulated in normal B cells, the pathway is often aberrantly activated in CLL. Disease course differs between U-CLL and M-CLL, where U-CLL is strongly associated with ZAP70 overexpression [116]. ZAP70 is a protein tyrosine kinase that plays an important role in the functioning of the immune system, including in the development and activation of B lymphocytes [117]. In CLL cell BCR signaling, the function of ZAP70 is enhancing signal transduction, which may contribute to an aggressive clinical course [118]. Other tyrosine kinases in BCR signaling components also include SYK and LYN, which have been shown to be upregulated in CLL at the protein level [119, 120]. The BCR signaling pathway includes or interacts with proteins with many other functions, such as integrins, adhesion molecules and cell cycle regulators. Due to its multitude of roles in CLL, BCR signaling proteins have been proposed as targets for the development of future therapeutic approaches [116]. In some cases, targeted therapies for BCR signaling have replaced chemoimmunotherapy [121], while next generation targeted therapies are still being investigated [122].

The NF-κB pathway regulates many processes, including both innate and adaptive immunity [123]. MYD88 participates in NF-κB signaling via TLR/IL-1R signaling, which are key elements in the immune response [124]. Changes in the protein have been identified as hallmarks of CLL and other malignancies [125]. Another affected pathway is the PI3K-AKT signaling pathway, which influences the regulation of the cell cycle, cellular quiescence, protein synthesis, proliferation, apoptosis and survival [126]. PI3K signaling governs BCR-dependent CLL cell proliferation and its inhibitors, such as idelalisib, have been approved for treatment, while novel inhibitors are still being developed [127]. Dysregulation of NOTCH1 signaling has also been associated with CLL, specifically enhanced survival of CLL cells [128]. Song et al. have also highlighted epiproteomics as an important and developing field in the understanding of drug resistance in cancers [129], making it a potentially interesting approach for future CLL research.

## Metabolomics, glycomics and lipidomics

Metabolomic studies provide useful information on the inner workings of a cell observing changes in metabolites. In cancer research, metabolomic studies have contributed to understand disease mechanisms and highlight potential therapeutic targets [130]. Piszcz et al. [131] studied metabolic indicators with liquid chromatography-mass spectrometry in order to determine if metabolites can discriminate between disease statuses. Six out of ten metabolites were found to be significantly increased in patients with aggressive CLL compared to indolent CLL and controls. Linoleamide and various acylcarnitine levels were significantly increased in aggressive CLL patients. Meanwhile, acetylcarnitine and hexannoylcarnitine were distinguishable markers for indolent CLL and healthy controls [131]. While metabolomics is concerned with metabolites, glycomics is the systematic study of carbohydrate systems. However, glycomic studies in the field of CLL research are limited, though Chen et al. [132] found a 2-fold difference in the GlcNAc-6-0-sulfated α2-6-sialyl LacNAc between CLL cells and healthy CD19+ lymphocytes [132].

The field of lipidomics is focuses on the study of cellular lipids, their functions and their role in metabolic pathways. A lipid analysis of glycolipids and sphingolipids highlighted large differences between U-CLL compared to M-CLL. The glucosylceramide to ceramide (Glu/Cer) ratio in U-CLL was increased by 441% compared to M-CLL. Compared to healthy controls, CLL cells displayed a 360% increased Glu/Cer ratio [133]. As these are important components of the cell membrane, it has been suggested that changes in membrane composition could contribute to chemotherapy resistance by reducing permeability [134]. The mevalonate pathway, responsible for the production of steroid molecules, also contributes to the control of CLL cell replication [135]. Simvastatin, which inhibits HMG-CoA reductase in the mevalonate pathway, decreases CLL cell proliferation and induces their apoptosis in vitro, and has been proposed as a treatment option either as a single agent or in combination with purine analogs [136].

## Multi-omics approaches

While individual omics levels may focus exclusively on a single type of cellular component or process, the goal of multi-omics is to integrate omics data from multiple levels in order to identify novel biomarkers, associations and potential treatment targets. Bloehdorn et al. [137] have characterized molecular subgroups of CLL based on genomic instability (GI) and activation of epithelial-mesenchymal-transition (EMT)-like programs, further subdivided into inflammatory and non-inflammatory subtypes [137]. GI-CLL have a disrupted DNA damage response while EMT-CLL exhibits high genomic stability. It has been suggested that the differences between the identified CLL subgroups, along with future assessments based on disease subtypes, may elucidate the therapeutic potential of targeting a combination of disease factors. A proposed example would be targeting BCL2 and PRMT5 or XPO1 alongside anti-CD20 monoclonal antibodies for GI-CLL cases [137].

Multi-omics approaches have also been used to assess CLL chemoresistance. The results of a single-cell multi-omics study by Thijssen et al. [138] suggest that venetoclax resistance in CLL is multi-layered. This is in part attributed to mutations in the BCL2 family, which act as apoptosis regulators. Upregulation of *MCL1* was also detected, as well as activation of the NF-κB pathway, which occurred during venetoclax therapy. It is also suggested that *MCL1* could be a direct transcriptional target of the pathway [138]. Another single-cell multi-omics study, by Hirayama et al. [139], investigated the influence of IGHV mutation status on CAR-T cell therapy. Long-term follow-ups were conducted and the results show that, in U-CLL, CD19 CAR-T cell immunotherapy is associated with durable remissions for high-risk cases of CLL [139].

An analysis was conducted on multiple data types: somatic mutations, CNVs, DNA methylation, RNA expression and ex vivo drug response phenotypes [140]. Data from 217 CLL tumor samples was analyzed with the Multi-Omics Factor Analysis (MOFA) method, which aims to find major axes of variation in tabular datasets. Seven factors were identified with this method, which were then tested for association with time-to-treatment (TTT) and overall survival (OS). Among these factors, factor 4 (F4) was most significant and higher F4 values were associated with shorter lymphocyte doubling time and worse outcomes [140]. F4 was also associated with multiple genomic aberrations. Clinical relevance was validated on independent datasets, totaling 547 treatment-naïve CLL samples. According to the authors, this method of classification is unlikely to be optimal and suggest it should be taken as a proof of concept, requiring further refinement by exploring various sets of biomarkers [140].

A study on both human and murine CLL cells showed that translation inhibition is a valuable strategy to block the translation of several oncogenic pathways including the MYC oncogene, thus controlling CLL development. Largeot et al. used FL3, a synthetic flavagline that acts as a prohibitin-binding drug, and used a multi-omic analysis on CLL patient samples and cell lines treated with FL3. The analysis revealed a decreased translation of the MYC oncogene as well as proteins involved in cell cycle and metabolism. The authors showed that high expression of translation initiation-related genes and prohibitin genes correlated with poor outcomes for CLL patients. Additionally, the authors demonstrated that the inhibition of MYC was responsible for major metabolism reprogramming. Furthermore, CLL development was controlled by translation inhibition both alone and combined with immunotherapy [141]. The multi-omics studies included in this review and the -omics levels analyzed therein are presented in Table 1.

**Table 1:** Multi-omics studies included in the present review and the omics levels included in their methodologies.

Omics level/ authors	Genomics	Epigenomics	Transcriptomics	Proteomics	Metabolomics	Single-cell multi-omics
Bloehdorn et al. [137]	✔	✔	✔	✔		
Thijssen et al. [138]	✔		✔			✔
Hirayama et al. [139]	✔		✔	✔		✔
Lu et al. [140]	✔	✔	✔			✔
Largeot et al. [141]			✔	✔	✔	

## The case of Richter transformation

RT is a severe complication of CLL or small lymphocytic lymphoma (SLL), where it transforms into a very aggressive large B cell lymphoma with outcomes much more severe than CLL. By one estimate the median time from CLL/SLL diagnosis to RT development is approximately 4 years, while median overall survival is 10 months [142]. There are many variants of RT, with the two most common being diffuse large B cell lymphoma (RT-DLBCL) and Hodgkin’s lymphoma (RT-HL) [143]. RT shares risk factors with CLL and SLL, however distinct markers have been identified on multiple omics levels that separate it from its parent diseases.

### Genomics

Common genetic factors associated with RT are genetic lesions in *TP53* [144]*, CDKN2A* [145]*, c-MYC* [146]*, NOTCH1* [146] and *MGA* [147], where lesions in *TP53* are the most common, occurring in up to 60-80% of RT cases [144]. Interestingly, the results of a study by Fabbri et al. [146] suggest that *TP53* disruption, *CDKN2A* loss and *MYC*-activating events often coexist. A multivariate analysis showed that a lack of *TP53* disruption translated into a significant survival advantage, suggesting it is an important factor for RT [148]. Additionally, 11q deletion, chromosome 12 trisomy, unmutated IGHV status and absence of 13q deletion have been associated with an increased RT risk [149]. While many RT risk factors are genomic aberrations, a number of SNPs have been found to be associated with RT. The rs6449182 variant in *CD38* has been associated with RT [150], as well as variants in *BCL2* and *LRP4* [151].

### Epigenomics

RT shows a higher degree of methylation for genes with the H3K27me3 mark and PRC2 targets compared to the preceding CLL phase and untransformed CLL [152]. It also shows increased methylation in genes that are targets of TP53 and RB1 [152]. Methylation profiles investigated using principal component analysis (PCA) also showed that CLL-derived and DLBCL-like RT subgroups differ by methylation profile and that the overall RS displays a hypomethylated profile [153]. Beside DNA methylation, miRNAs have also been associated with RT. miR-21, miR-148b and miR-181b were shown to be upregulated in RT compared to CLL controls. miR-21, miR-24, miR-26a and miR-146b also showed differential expression at the time of RT diagnosis. A network analysis of these miRNAs showed that their targets were significantly enriched in pathways involved in cancer, immunity and inflammation [154]. B cell receptors (BCR) immunoglobulin stereotyped subset 8 is associated with a higher risk of RT. Tsagiopoulou et al. [54] report that this subset displays a distinct chromatin activation profile, similar to that found in U-CLLs developing into RT.

### Transcriptomics

CLL and RT display differences on many omics levels, which also extends to transcriptomics. Klintman et al. [155] compared the RNA expression levels between nodal CLL and tissue RT samples. They found that *KRAS* and *BRAF* were underexpressed in tissue RT samples compared to CLL and were also affected by deletions. However, *RAD52, POLRJ2, BRCA2* and *ATR* were downregulated and *PARP* and *FANCG* were overexpressed without being correlated with mutations in genes. Given the functions of these genes, the results suggest that DNA damage repair (DDR) mechanisms play an important role in RT [155]. The results of a longitudinal study by Nadeu et al. [156] show that the expression profiles of RT and CLL cells are highly different. Transcriptionally, CLL cells could be best categorized in three clusters – those categorized by differential expression of *CXCR4, CD27* or *MIR155HG* respectively. Conversely, RT heterogeneity was mainly related to proliferative capacities. A cluster of cells showed high *MKI67* and *PCNA* expression. Other RT clusters were characterized by the differential expression of *CCND2, MIR155HG* and *TP53INPI*. It is suggested that RT is transcriptionally and epigenetically reminiscent of the de novo DLBCL CLL subtype, which is characterized insensitivity to BCR inhibition and high oxidative phosphorylation (OXPHOS), which could explain rapid expansion of RT subclones under therapy of BCR inhibitors [156].

### Proteomics

Members of the protein kinase B (PKB) family, also known as Akt, are serine/threonine-specific protein kinases that play roles in regulating apoptosis, cell proliferation, transcription, metabolism and other cellular functions. At the proteomic level, Akt phosphorylation has been shown to be associated with RT. Compared to CLL, RT has increased frequency and intensity of Akt phosphorylation. Additionally, CLL samples from high RT risk patients showed significantly increased Akt phosphorylation. It is suggested that Akt initiates RT development by including Notch1 signaling in B cells [157]. However, a principal component analysis of proteomic profiles of CLL cases showed that RT cases and CLL cases were intermixed, suggesting that the proteomics of circulating CLL cells that have undergone RT have not changed significantly [20].

### Metabolomics, glycomics and lipidomics

Metabolically, CLL cells are highly glycolytic, though not to the same degree as DLBCL cells [158]. When neoplastic cells exhibit high FDG uptake during PET scans, it is strongly suggestive of RT, however tissue biopsies should still be preferred for diagnosing RT [159]. In murine models, CLL and RT B cells had higher levels of cellular and mitochondrial reactive oxygen species (ROS) than control B cells. Additionally, RT B cells had higher cellular ROS than CLL cells. RT cells also showed a high usage of TCA cycle substrates [160]. Results from a study on murine models showed that the *MGA/MYC/NME1* axis drives RT via the accumulation of ROS and increased mitochondrial OXPHOS. In murine models targeting this axis provided therapeutic benefits, suggesting that it could be a potential novel target for RT treatment [160].

### Multi-omics

Multi-omics in the study of RT has not yet been widely employed. Broséus et al. [153] have characterized human RT samples by genome-wide DNA methylation and whole-transcriptome profiling, developing DNA methylation and transcription-based classifiers. The classification approach can robustly identify phenotypes similar to RT, which could be clinically significant. Additionally, the integration of DNA methylation and transcriptomic data has highlighted the involvement of EZH2 and Wnt pathways, as well as PI3kinase/Akt and IGFR1 signaling as mechanisms that could contribute to RT and chemotherapy resistance [153].

## Summary

Genes associated with CLL and included in the present review are listed in Table 2. Chromosome locations were obtained from the Ensembl genome browser [77].

**Table 2:** Genes associated with CLL and included in the present review.

Gene symbol	Gene name [HGNC gene ID]	Chr.	Regulation
*MIR200A*	microRNA 200a [HGNC:31578]	1	Downregulated by *HULC*
*MIR34A*	microRNA 34a [HGNC:31635]	1	Downregulated
*ARID1A*	AT-rich interaction domain 1A [HGNC:11110]	1	Deletions and insertions
*HDAC1*	histone deacetylase 1 [HGNC:4852]	1	Identified as a potential treatment target
*MCL1*	MCL1 apoptosis regulator, BCL2 family member [HGNC:6943]	1	Upregulated; potential differential effect on CLL depending on isoform.
*FCGR2B*	Fc gamma receptor IIb [HGNC:3618]	1	Upregulated
*MIR181B1*	microRNA 181b-1 [HGNC:31550]	1	Downregulated
*ATP2B4*	ATPase plasma membrane Ca2+ transporting 4 [HGNC:817]	1	Overexpressed
*LAX1*	lymphocyte transmembrane adapter 1 [HGNC:26005]	1	Overexpressed
*RASSF5*	Ras association domain family member 5 [HGNC:17609]	1	Upregulated in patients with low miR-15a/miR-16-1 expression
*PIGR*	polymeric immunoglobulin receptor [HGNC:8968]	1	Overexpressed
*GDF7*	growth differentiation factor 7 [HGNC:4222]	2	Positive correlation with the expression of *CRNDE* and *AC12065.7*
*XPO1*	exportin 1 [HGNC:12825]	2	A proposed target for combined therapy
*ZAP70*	zeta chain of T cell receptor associated protein kinase 70 [HGNC:12858]	2	Upregulated in CLL cases with hypomethylated DNA; upregulated in Binet stage A and B
*SF3B1*	splicing factor 3b subunit 1 [HGNC:10768]	2	Splice site alterations; associated with fludarabine refractoriness; no differential expression detected
*CTLA4*	cytotoxic T-lymphocyte associated protein 4 [HGNC:2505]	2	Affected by H3K27 acetylation
*MYD88*	MYD88 innate immune signal transduction adapter [HGNC:7562]	3	Single nucleotide variant
*CLEC3B*	C-type lectin domain family 3 member B [HGNC:11891]	3	Suggested as a potential novel therapeutic target
*SETD2*	SET domain containing 2, histone lysine methyltransferase [HGNC:18420]	3	Deletions
*CDC25A*	cell division cycle 25A [HGNC:1725]	3	Targeted by the *miR-16* family
*LRIG1*	leucine rich repeats and immunoglobulin like domains 1 [HGNC:17360]	3	Affected by the *miR-15a*/*miR-16-1* cluster
*TP63*	tumor protein p63 [HGNC:15979]	3	Upregulated in CLL cases with hypomethylated DNA
*CD38*	CD38 molecule [HGNC:1667]	4	No differential expression detected; variant associated with Richter transformation
*LEF1*	lymphoid enhancer binding factor 1 [HGNC:6551]	4	Affected by H3K27 acetylation
*FBXW7*	F-box and WD repeat domain containing 7 [HGNC:16712]	4	Missense, nonsense, indels and splice site variants
*FAT1*	FAT atypical cadherin 1 [HGNC:3595]	4	Associated with fludarabine refractoriness
*HDAC3*	histone deacetylase 3 [HGNC:4854]	5	Identified as a potential treatment target
*HULC*	hepatocellular carcinoma upregulated long non-coding RNA [HGNC:34232]	6	Upregulated in CLL; downregulates *miR-372* and *miR-200a-3p*
*TNF*	tumor necrosis factor [HGNC:11892]	6	Methylated
*MAPK13*	mitogen-activated protein kinase 13 [HGNC:6875]	6	Identified as a potential treatment target
*NFKBIE*	NFKB inhibitor epsilon [HGNC:7799]	6	Deletions and other variants
*FOXO3*	forkhead box O3 [HGNC:3821]	6	Overexpressed
*CCN2*	cellular communication network factor 2 [HGNC:2500]	6	Overexpressed
*MIR148A*	microRNA 148a [HGNC:31535]	7	Higher expression associated with fludarabine resistant CLL
*EZH2*	enhancer of zeste 2 polycomb repressive complex 2 subunit [HGNC:3527]	7	Overexpressed; highlighted to potentially contribute to RT and chemotherapy resistance
*LYN*	LYN proto-oncogene, Src family tyrosine kinase [HGNC:6735]	8	Upregulated
*MYC*	MYC proto-oncogene, bHLH transcription factor [HGNC:7553]	8	Associated with BCR signaling; associated with Richter transformation
*PAX5*	paired box 5 [HGNC:8619]	9	Variants in enhancer region
*SYK*	spleen associated tyrosine kinase [HGNC:11491]	9	Upregulated
*MIR181B2*	microRNA 181b-2 [HGNC:31551]	9	Downregulated
*NOTCH1*	notch receptor 1 [HGNC:7881]	9	Deletions, substitutions and other variants; no dysregulation found; associated with Richter transformation
*MAPK8*	mitogen-activated protein kinase 8 [HGNC:6881]	10	Identified as a potential treatment target
*BLNK*	B cell linker [HGNC:14211]	10	Upregulated
*CASC2*	cancer susceptibility 2 [HGNC:22933]	10	Proposed as a novel therapeutic target or clinical marker
*CTBP2*	C-terminal binding protein 2 [HGNC:2495]	10	Methylated
*WEE1*	WEE1 G2 checkpoint kinase [HGNC:12761]	11	Identified as a potential treatment target
*MIR192*	microRNA 192 [HGNC:31562]	11	Downregulated
*NEAT1*	nuclear paraspeckle assembly transcript 1 [HGNC:30815]	11	Induced by a functional P53; induction correlated with apoptosis after DNA damage
*MALAT1*	metastasis associated lung adenocarcinoma transcript 1 [HGNC:29665]	11	Overexpressed
*CCND1*	cyclin D1 [HGNC:1582]	11	Upregulated
*BIRC3*	baculoviral IAP repeat containing 3 [HGNC:591]	11	Single nucleotide variants and deletions
*ATM*	ATM serine/threonine kinase [HGNC:795]	11	Downregulated in Binet stage A and B
*MIR34B*	microRNA 34b [HGNC:31636]	11	Located in a commonly deleted region; frequently downregulated
*MIR34C*	microRNA 34c [HGNC:31637]	11	Located in a commonly deleted region; frequently downregulated
*CXCR5*	C-X-C motif chemokine receptor 5 [HGNC:1060]	11	Upregulated in Binet stage A, downregulated in Binet stage B; proposed as a potential novel treatment target or marker
*CHEK1*	checkpoint kinase 1 [HGNC:1925]	11	Targeted by the *miR-16* family
*C1S*	complement C1s [HGNC:1247]	12	Proposed as a potential novel treatment target or marker
*HDAC7*	histone deacetylase 7 [HGNC:14067]	12	Identified as a potential treatment target
*RNF41*	ring finger protein 41 [HGNC:18401]	12	Affected by the *miR-15a*/*miR-16-1* cluster
*STAT2*	signal transducer and activator of transcription 2 [HGNC:11363]	12	Expression linked to patient response to kinase inhibitors
*BAZ2A*	bromodomain adjacent to zinc finger domain 2A [HGNC:962]	12	Affected by the *miR-15a*/*miR-16-1* cluster
*E2F7*	E2F transcription factor 7 [HGNC:23820]	12	Targeted by the *miR-16* family
*TMCC3*	transmembrane and coiled-coil domain family 3 [HGNC:29199]	12	Overexpressed
*CKAP4*	cytoskeleton associated protein 4 [HGNC:16991]	12	Overexpressed
*CRY1*	cryptochrome circadian regulator 1 [HGNC:2384]	12	Proposed as a potential novel treatment target or marker
*RB1*	RB transcriptional corepressor 1 [HGNC:9884]	13	Deleted in some CLL cases; targets of RB1 show increased methylation in Richter transformation cases
*DLEU2*	deleted in lymphocytic leukemia 2 [HGNC:13748]	13	Affected by 13q14 deletion
*MIR16-1*	microRNA 16-1 [HGNC:31545]	13	Downregulated; located on the commonly deleted 13q14
*MIR15A*	microRNA 15a [HGNC:31543]	13	Downregulated; located on the commonly deleted 13q14
*DLEU1*	deleted in lymphocytic leukemia 1 [HGNC:13747]	13	Affected by 13q14 deletion
*MIR17HG*	miR-17-92a-1 cluster host gene [HGNC:23564]	13	Associated with CLL; overexpressed in murine models
*PRMT5*	protein arginine methyltransferase 5 [HGNC:10894]	14	Proposed as a target for combined treatment
*TCL1A*	TCL1 family AKT coactivator A [HGNC:11648]	14	Targeted by *miR-29* and *miR-181*
*MIR342*	microRNA 342 [HGNC:31778]	14	Upregulated
*CHD2*	chromodomain helicase DNA binding protein 2 [HGNC:1917]	15	Altered splice sites, frameshift and nonsense variants
*HMOX2*	heme oxygenase 2 [HGNC:5014]	16	Identified as a potential treatment target
*RBL2*	RB transcriptional corepressor like 2 [HGNC:9894]	16	Associated with BCR signaling
*CRNDE*	colorectal neoplasia differentially expressed [HGNC:37078]	16	Promoter is hyperhemtylated; expression correlated with *GDF7* and *IRX5* expression
*IRX5*	iroquois homeobox 5 [HGNC:14361]	16	Expression correlated with *CRNDE* and *AC012065.7*
*URAHP*	urate (hydroxyiso-) hydrolase, pseudogene [NCBI Acc:100130015]	16	Proposed as a potential novel treatment target or marker
*SCARF1*	scavenger receptor class F member 1 [HGNC:16820]	17	Methylated
*TP53*	tumor protein p53 [HGNC:11998]	17	Deletions, missense variants, frameshift variants and others, genetic lesions associated with Richter transformation
*IKZF3*	IKAROS family zinc finger 3 [HGNC:13178]	17	More commonly altered in unmutated IGHV CLL
*MIR21*	microRNA 21 [HGNC:31586]	17	Overexpressed; associated with fludarabine resistant CLL; upregulated in RT compared to CLL
*ARHGAP27P2*	Rho GTPase activating protein 27 pseudogene 2 [HGNC:53771]	17	Proposed as a novel therapeutic target or clinical marker
*MIR338*	microRNA 338 [HGNC:31775]	17	Downregulated
*B3GNTL1*	UDP-GlcNAc:betaGal beta-1,3-N-acetylglucosaminyltransferase like 1 [HGNC:21727]	17	Methylated
*GATA6-AS1*	GATA6 antisense RNA 1 (head to head) [HGNC:48840]	18	Promoter is methylated
*BCL2*	BCL2 apoptosis regulator [HGNC:990]	18	Affected by H3K27 acetylation
*NFATC1*	nuclear factor of activated T cells 1 [HGNC:7775]	18	Upregulated
*RPS15*	ribosomal protein S15 [HGNC:10388]	19	Single nucleotide variants, multiple nucleotide variants
*FUT5*	fucosyltransferase 5 [HGNC:4016]	19	Proposed as a potential novel treatment target or marker
*CLEC17A*	C-type lectin domain containing 17A [HGNC:34520]	19	Overexpressed
*CCNE1*	cyclin E1 [HGNC:1589]	19	Targeted by the *miR-16* family
*MIR372*	microRNA 372 [HGNC:31786]	19	Downregulated by *HULC*
*SAMHD1*	SAM and HD domain containing deoxynucleoside triphosphate triphosphohydrolase 1 [HGNC:15925]	20	Deletions and other variants
*MIR155*	microRNA 155 [HGNC:31542]	21	Overexpressed; higher expression in cases with incomplete treatment response
*HMOX1*	heme oxygenase 1 [HGNC:5013]	22	Identified as a potential treatment target
*MID1IP1*	MID1 interacting protein 1 [HGNC:20715]	X	Proposed as a potential novel treatment target or marker
*MIR222*	microRNA 222 [HGNC:31602]	X	Higher expression associated with fludarabine resistant CLL
*BTK*	Bruton tyrosine kinase [HGNC:1133]	X	Upregulated in the BCR pathway; no differential expression between Binet stage A and stage B
*DKC1*	dyskerin pseudouridine synthase 1 [HGNC:2890]	X	Underexpressed; underexpression associated with poor survival following treatment

While the list of CLL-associated genes in the present review is not yet complete, it can serve as a basis for future research with an expanded scope.

## Future directions

Extensive research has been done on CLL and has yielded large quantities of data on contributing factors. However, these factors, when taken individually, may not accurately predict disease course nor optimal treatment choices. The challenge now is the successful combining of available data in a manner that can stratify patients into groups with practical applications, so that clinicians can prescribe optimal treatment regimens. For this purpose, machine learning approaches may be successful in assisting researchers in grouping disease subtypes based on what treatment may be most effective.

While recent advances in treatment, such as ibrutinib and venetoclax therapy, have improved patient outlook, cases such as RT have yet to be addressed. Novel treatment targets, as well as therapeutics that can target them, are therefore a high priority. Similarly, further investigation into combinatorial treatment with different therapeutics may yield beneficial results for patients with certain CLL types.

## Conclusions

Thus far, most studies have focused on a single-omics level yielding significant findings and enabling the development of treatment options. However, while valuable, these treatments may not provide reliable solutions for all CLL types, such as RT cases. Integrated omics, or multi-omics, offer a more comprehensive approach to understanding diseases providing novel insights into their underlying pathomechanisms. As multi-omics approaches are well-suited for studying complex diseases like CLL, they are well suited for identification of targets for new or combined treatments. Moreover, this approach has the potential to aid clinicians in making improved treatment decisions by identifying disease subgroups, leading to improved outcomes for patients.
